# Editorial: Chemo-Resistance in Gastrointestinal Cancers

**DOI:** 10.3389/fonc.2022.821212

**Published:** 2022-01-28

**Authors:** Xia Li, Dong-Hua Yang, Prathibha Ranganathan

**Affiliations:** ^1^Research Center for Biomedical Information Technology, Shenzhen Institutes of Advanced Technology, Chinese Academy of Sciences, Shenzhen, China; ^2^College of Pharmacy and Health Sciences, St. John’s University, Queens, NY, United States; ^3^Centre for Human Genetics (CHG), Bengaluru, India

**Keywords:** gastric cancer, drug-resistance, mechanism, targets, biomarker

Gastrointestinal (GI) cancers are a group of diseases originating from different organs of the gastro-intestinal tract, with very high ranking of cancer incidence and mortality (1). Chemotherapy is widely used in GI cancer treatment, and is the preferred choice of treatment for patients who cannot undergo surgical resection or in patients with advanced metastases (2). Patients with GI cancer can benefit from chemotherapy including these commonly used chemotherapeutic agents, adriamycin, platinum drugs, 5-fluorouracil, vincristine, and paclitaxel. However, it is often observed that there is poor or no response to chemotherapy, and even in patients who respond well during primary treatment, the long-term results are disappointing. The development of chemo-resistance is a major obstacle in management of GI cancers. Various causes of drug resistance have been identified, which include inactivation of apoptosis signaling pathways, loss of cell cycle checkpoint control, accelerated cell proliferation and autophagy flux, enhanced DNA damage repair capacity, cancer stem cells, as well as epithelial-mesenchymal transition (3–5). Unfortunately, the precise mechanism of adaptive changes during development of chemo-resistance in GI cancers is still unclear. An insight into the mechanisms of chemo-resistance in GI cancers may help to devise better and personalized treatment strategies.

This Research Topic was aimed at bringing together clinical and basic scientists and to address the problem of chemo-resistance from multiple perspectives. True to its aim, this collection has a variety of articles including original research, review of literature and case reports. They cover areas such as signaling pathways, non-coding RNAs, cancer stem cells, and biomarkers to predict tumor outcome.

The Mitogen Activated Protein Kinase (MAPK) pathway is one of the most important signaling pathways involved in normal cellular processes, and plays a key role in the development and progression of cancer (6). Once activated, MAPK exerts an important role in converting extracellular stimuli into a wide range of cellular responses. Increasing evidences support its role in response to chemotherapeutic agents (7, 8). Upon stimulation, ERK1/2 signaling could decrease the sensitivity to sorafenib in hepatocellular carcinoma cells (9). Understanding the main effector genes along with downstream pathways can identify potential therapeutic targets. In this special guest edition, Li B. et al. discovered that phosphorylation of endogenous ERK1/2 could be stimulated by GCDA (Glycochenodeoxycholate) in hepatocellular carcinoma cells. Disruption of the effect of GCDA by blocking phosphorylation and nuclear accumulation of ERK1/2 could be potentially a mode of managing GCDA-related liver cancer and chemo-resistance. In addition, Gao et al. demonstrated that CIDEA expression promoted the chemosensitivity of esophageal cancer cells to cisplatin by activation of JNK. This study further suggests a tumor suppressor role for CIDEA as well and opens a possibility of using this as a target for combinatorial therapy. A study by Li Y. et al. reported a case of advanced colorectal cancer who was successfully treated using anti-EGFR drugs (cetuximab) in combination with anti-VEGF agents (fruquintinib) after development of resistance to chemotherapy. The mechanism underlying the success of this combinatorial therapy however needs further investigation.

In GI cancers, multiple deregulated noncoding RNAs (ncRNAs), including miRNAs and lncRNAs, play pivotal roles in the development of chemo-resistance (10, 11). In this regard, Qian et al. found that miR-454-3p was significantly up-regulated in oxaliplatin-resistant colorectal cancer cells and miR-454-3p promoted oxaliplatin resistance by targeting PTEN and activating the AKT signaling pathway. PI3K/AKT/mTOR pathway is also one of the most important signaling pathways involved in chemo-resistance in many human cancers (12). The contribution by Chen et al. demonstrated that HOXA13 overexpression increased 5-fluorouracil resistance in gastric cancer cells, and the expression of HOXA13 was directly suppressed by miR-139-5p. Bai et al. demonstrated the role of lncRNA AC007639.1 in chemo-resistance of hepatocellular carcinoma using a combination of bioinformatics and experimental approaches. These findings highlight targeting ncRNAs may act as a potential therapeutic strategy for reducing resistance to chemotherapy. Given the importance of ncRNAs in cancer, systematic exploration of the crosstalk with other molecular players should aid in a better understanding of their roles during the process of development of drug resistance of GI cancer patients.

A study by Liu et al. demonstrated the role of cancer stem cells as well as epithelial-mesenchymal transition (EMT) responsible for the development of chemo-resistance in esophageal cancer. Cancer stem cells have been reported in different GI cancers and are thought to be responsible for tumor initiation, metastasis, and drug resistance (13). The role of EMT in cancer drug resistance has long been suggested. It is worth noting that tumor microenvironment (TME) is also a factor mediating EMT-driven drug resistance, and the interactions of cancer cells with TME are crucial in EMT and drug resistance (14). Therefore it would be necessary to delineate detailed relationships of cancer cells and TME during chemo-resistance, such as the spatial locations of cancer and immune cells in the tumor tissues and specific ligand-receptor interactions between them.

Identification of biomarkers could improve diagnosis, prognosis, prediction of recurrence and treatment response. Many studies have contributed to the discovery of prognostic biomarkers, but the clinical need lies much more in the predictive biomarkers which would aid in deciding therapeutic approaches to improve patient outcomes. In this context, Sun et al. comprehensively reviewed progresses on the sensitivity prediction of neoadjuvant chemoradiotherapy for GI cancers in the aspects of microRNAs, metabolic enzymes, exosomes, other biomarkers, inflammatory indicators, and imageological assessments. Notably, a recent review has a special focus on the immune markers from TME and discusses their predictive roles on response to cytotoxic chemotherapy in colorectal cancer (15). The emerging move to the discovery and establishment of biomarkers from TME has the potential to develop more robust biomarkers for therapy benefit and resistance of chemotherapy. Alternatively, prediction of drug sensitivity through bioinformatics and computational biology could represent one efficient way to manage large and complex data sets, and provide prior information in the theoretical guidance. Innovative and advanced biomarker prediction approaches, together with large cohorts validation, should be more extensively designed and conducted in the near future.

Altogether, the articles collected in this Research Topic provide a series of insightful sets of data to better understand molecular events involved in the development of chemo-resistance in GI cancers. These findings offer implications in potential therapeutic targets identification, and provide insight on further drug-resistance research. It is our hope that this effort would pave the way for more inter-disciplinary work with the goal of better management of GI cancers.

## Author Contributions

All authors listed have made a substantial, direct and intellectual contribution to the work, and approved it for publication.

## Conflict of Interest

The authors declare that the research was conducted in the absence of any commercial or financial relationships that could be construed as a potential conflict of interest.

## Publisher’s Note

All claims expressed in this article are solely those of the authors and do not necessarily represent those of their affiliated organizations, or those of the publisher, the editors and the reviewers. Any product that may be evaluated in this article, or claim that may be made by its manufacturer, is not guaranteed or endorsed by the publisher.
